# Socio-occupational factors and problematic internet use: indirect associations involving negative affect and impulsivity in a predominantly urban, highly educated Romanian adult sample

**DOI:** 10.3389/fpsyg.2026.1940887

**Published:** 2026-10-30

**Authors:** Cătălina T. Țeglet, Alin-Sebastian Godeanu, Vasile Constantin

**Affiliations:** Department of Applied Psychology and Psychotherapy, Faculty of Psychology and Educational Sciences, University of Bucharest, Bucharest, Romania

**Keywords:** I-PACE model, negative affect, problematic internet use, serial indirect effects, work addiction

## Abstract

**Background:**

Problematic internet use (PIU) has been associated with emotional distress, impulsivity, and occupational factors, but their interrelations remain insufficiently examined in adult populations. Guided by the Interaction of Person–Affect–Cognition–Execution (I-PACE) model, this study examined socio-occupational, emotional, and self-regulatory correlates of PIU.

**Objective:**

We investigated whether perceived social support, work engagement, and work addiction were indirectly associated with PIU through negative affect and trait impulsivity within a theory-specified sequence.

**Method:**

A cross-sectional online survey was completed by 215 Romanian adults from a predominantly urban and highly educated convenience sample (59.5% women; *M*_age_ = 37.11, SD = 9.16). Participants completed Romanian-language measures of perceived social support, work engagement, work addiction, depressive symptoms, trait anxiety, impulsivity, and PIU. Pearson correlations, hierarchical regression, and serial indirect-effect analyses (PROCESS Model 6; 10,000 bootstrap samples) were conducted. Primary models controlled for age, gender, and daily personal internet use; supplementary analyses examined mutual adjustment among the socio-occupational predictors and alternative mediator ordering.

**Results:**

PIU was negatively associated with perceived social support and work engagement and positively associated with work addiction, depressive symptoms, trait anxiety, and impulsivity. The final regression model explained 45.5% of PIU variance. In all three primary models, the indirect associations through negative affect and through the serial sequence involving negative affect and impulsivity were significant, whereas those through impulsivity alone were not. The theory-specified serial associations remained significant after mutual adjustment. Under the reversed mediator ordering, the serial indirect association was significant for perceived social support and work engagement, but not for work addiction. Work addiction retained a significant direct association with PIU.

**Conclusion:**

The findings were consistent with selected components of the I-PACE model. Negative affect represented a prominent indirect pathway, while work addiction showed both direct and indirect associations with PIU. Because all variables were measured concurrently, the findings do not establish temporal precedence or causal mechanisms but may inform further research in comparable adult samples.

## 1 Introduction

### 1.1 General context

The internet has become embedded in everyday life, supporting communication, occupational activities, access to information, and leisure. For most individuals, frequent internet use is functional and does not necessarily indicate psychological impairment. Difficulties emerge when online activity becomes poorly controlled, cognitively preoccupying, and persistent despite adverse consequences for psychological, social, or occupational functioning. Although this phenomenon has been described using terms such as internet addiction, internet use disorder, and compulsive internet use, problematic internet use (PIU) provides a broader conceptualization of maladaptive internet use along a continuum rather than as a discrete diagnostic category (Fineberg et al., 2022; Moretta et al., 2022). PIU is therefore distinguished from time spent online alone and is defined primarily by diminished control, persistent preoccupation, functional impairment, and continued engagement despite negative consequences (Brand et al., 2019; Davis, 2001; Kardefelt-Winther, 2014). This distinction is particularly important in contemporary adult life, where extensive internet use may reflect legitimate occupational and personal demands rather than problematic behavior.

### 1.2 PIU in adults and the occupational context

Most research on PIU has focused on adolescents, university students, or specific online activities such as gaming and social networking. Comparatively less attention has been paid to adults, although internet use in this population is closely integrated with occupational responsibilities, remote work, information management, and continuous digital communication. These demands can blur the boundaries between professional and personal life and make it particularly important to distinguish necessary or intensive internet use from patterns characterized by diminished control and functional impairment (Ghislieri et al., 2022; Vayre and Vonthron, 2019). Examining PIU in adults therefore requires consideration not only of time spent online but also of the social, emotional, and occupational contexts in which internet use occurs.

Two contrasting forms of work investment may be relevant to PIU. Work engagement, characterized by vigor, dedication, and absorption, reflects a positive and fulfilling relationship with work. As a positive occupational resource, work engagement may be associated with lower emotional distress and, consequently, with less reliance on online activities for compensation or avoidance. Work addiction, by contrast, involves excessive and compulsive involvement in work, an internal pressure to continue working, and difficulty disengaging from occupational demands (Schaufeli et al., 2009). These characteristics may coexist with emotional strain and poorly regulated behavioral patterns, making work addiction a plausible occupational correlate of PIU. Importantly, work addiction and PIU remain conceptually distinct: the former concerns compulsive involvement in work, whereas the latter concerns impaired control over internet use.

Perceived social support represents a complementary interpersonal resource. Supportive relationships may buffer psychological distress and reduce the tendency to seek emotional relief or social compensation primarily through online environments. Lower perceived support, lower work engagement, and higher work addiction may therefore show different patterns of association with PIU, while also being related to the emotional states associated with problematic use. Examining these constructs together may clarify whether socio-occupational resources and vulnerabilities are associated with PIU beyond demographic characteristics and the amount of time spent online.

### 1.3 The I-PACE model

The Interaction of Person–Affect–Cognition–Execution (I-PACE) model provides a useful framework for examining how distal psychosocial factors are associated with problematic internet use (Brand et al., 2025). Within this framework, social and occupational resources and vulnerabilities may be linked to problematic online behavior through proximal affective and self-regulatory processes. In the present study, negative affect was conceptualized as a broad transdiagnostic dimension of emotional distress reflected in the shared component of depressive symptoms and trait anxiety. This conceptualization is consistent with the tripartite model, according to which depression and anxiety share a general negative-affect dimension while retaining distinct features, including low positive affect in depression and physiological hyperarousal in anxiety (Clark and Watson, 1991). Depressive symptoms and trait anxiety were therefore treated as indicators of a shared affective vulnerability rather than as conceptually equivalent constructs. Within the theory-specified model, lower perceived social support and work engagement were expected to be associated with lower negative affect, whereas work addiction was expected to be associated with higher negative affect. Trait impulsivity was conceptualized as an indicator of diminished self-regulatory capacity that may be associated with PIU and may also be statistically related to negative affect within the proposed model. Within the I-PACE framework, affective responses are assumed to interact with executive-control processes during decision-making, and elevated negative affect may be associated with temporarily reduced inhibitory control and a greater expression of impulsive responding (Brand et al., 2025). This provided the theoretical rationale for specifying negative affect before impulsivity in the statistical model. However, because the BIS-11 assesses a relatively stable dispositional characteristic, the model did not assume that negative affect produces or changes trait impulsivity. Instead, trait impulsivity was treated as an indicator of self-regulatory vulnerability within a theory-specified statistical sequence. Because the present study was cross-sectional, this ordering was examined as a theory-informed statistical sequence rather than as an empirically established temporal or causal process.

#### 1.3.1 The present study

Although previous studies have documented associations between PIU, emotional distress, impulsivity, and work-related variables, relatively few have examined these factors within a single integrative model among adults. Moreover, the potential indirect associations involving negative affect and impulsivity within a theory-specified serial model have received limited empirical attention.

Therefore, the present study examined a theory-specified serial indirect-effect model involving perceived social support, work engagement, work addiction, negative affect, impulsivity, and problematic internet use. Guided by the I-PACE framework, we examined whether the associations between socio-occupational factors and PIU included indirect effects through negative affect and whether the proposed serial indirect pathway additionally involved impulsivity as a self-regulatory correlate. Because all variables were measured concurrently, the ordering of negative affect and impulsivity was theoretically specified and was not intended to establish temporal precedence or causal mechanisms. To achieve this overarching aim, three core hypotheses were formulated. The hypotheses were formulated prior to the statistical analyses on the basis of the I-PACE framework and the reviewed literature. However, they were not preregistered and should therefore be regarded as theory-informed rather than preregistered confirmatory predictions.

*Hypothesis 1* (Bivariate Associations): We hypothesized that PIU would be significantly associated with all investigated distal and proximal variables. Specifically, PIU was expected to negatively correlate with adaptive resources (perceived social support and work engagement) and positively correlate with maladaptive vulnerabilities (work addiction, depressive symptoms, trait anxiety, and impulsivity).

*Hypothesis 2* (Incremental Statistical Contribution): Beyond baseline socio-demographic characteristics (age and gender) and screen time indicators (personal time spent online), we hypothesized that distal psychosocial factors (social support, work engagement, work addiction), proximal emotional distress (negative affect), and cognitive-executive characteristics (impulsivity) would sequentially explain significant incremental proportions of variance in adult PIU scores.

*Hypothesis 3* (Theory-Specified Indirect Associations): We expected the associations between the socio-occupational variables and PIU to involve indirect associations through negative affect and trait impulsivity. More specifically:

- The associations of perceived social support and work engagement with PIU were expected to involve indirect associations through negative affect.
- Work addiction was expected to show a serial indirect association with PIU through negative affect followed by trait impulsivity. Across the models, the indirect associations through trait impulsivity alone were not expected to be statistically distinguishable from zero.

## 2 Methods

### 2.1 Participants and procedure

Participants were recruited using convenience sampling through professional and social networking platforms (e.g., Facebook messenger and WhatsApp). Data were collected between March and April 2026 using an anonymous online questionnaire administered via Google forms. Before participation, respondents provided informed consent after receiving information about the study objectives, the voluntary nature of participation, confidentiality, and their right to withdraw at any time. No financial or material incentives were offered. The questionnaire required approximately 15 min to complete.

Eligibility criteria included being at least 18 years of age, being an active internet user, and providing informed consent. Following data screening, no incomplete questionnaires, duplicate submissions, underage participants, or otherwise ineligible cases were identified. The final sample comprised 215 Romanian adults aged 18–60 years (59.5% women; *M*_age_ = 37.11, SD = 9.16). Because convenience sampling was used and recruitment occurred exclusively through online platforms, the sample should not be considered representative of the Romanian adult population.

An *a priori* power analysis conducted using G*Power 3.1 (Faul et al., 2009) indicated that a minimum sample of 107 participants was required to detect a medium effect size (f^2^ = 0.15) in a hierarchical multiple regression model including nine predictors (*α* = 0.05, power = 0.80). The final sample (*N* = 215) exceeded this requirement. However, this power analysis was designed specifically for the hierarchical regression and did not provide a sample-size justification for the serial indirect-effect models. The indirect effects were estimated using 10,000 bootstrap resamples and interpreted on the basis of their 95% confidence intervals, while recognizing that the sample may provide limited precision for estimating complex serial indirect effects.

Participants ranged across adulthood, with Most aged 30–44 years (*n* = 131, 60.9%), followed by 18–29 years (*n* = 49, 22.8%), 45–59 years (*n* = 32, 14.9%), and 60 years or older (*n* = 3, 1.4%). Most participants lived in urban areas (90.7%) and had completed higher education, with 52.6% holding postgraduate or doctoral degrees and 40.9% holding a bachelor’s or post-secondary degree. The majority were employed (80.9%), whereas 8.8% were self-employed or freelancers and 10.2% were outside the active workforce. Participants who were not currently employed were instructed to complete the UWES-9 and DUWAS-10 with reference to their most recent job. These participants were retained in all analyses.

Compared with national indicators, the present sample disproportionately represented urban, highly educated, employed, and younger adults. Urban residents comprised 90.7% of the sample, compared with 55.2% nationally (Agerpres, 2026), while 89.7% were employed or self-employed, compared with a national employment rate of 69.0% among adults aged 20–64 (Eurostat, 2026b). Furthermore, 52.6% held postgraduate or doctoral qualifications, whereas approximately 19% of Romanian adults aged 25–64 held a tertiary qualification (Eurostat, 2026a; OECD, 2025). An additional 40.9% selected a combined bachelor’s or post-secondary category, preventing these distinct educational levels from being examined separately (UNESCO Institute for Statistics, 2012). Finally, the sample had a mean age of 37.11 years and only 1.4% were aged 60 or older, compared with a national median age of 44.3 years and 20.3% of the population aged 65 or older (Agerpres, 2025; Agerpres, 2026).

Regarding personal (non-work-related) internet use, 44.2% of participants reported spending 1–3 h online per day, 31.2% reported 4–5 h, 8.8% reported 6–7 h, 8.4% reported more than 8 h, and 7.4% reported less than 1 h. the most frequently reported primary online activity was social communication (46.0%), followed by entertainment (42.3%) and utilitarian or informational purposes (11.6%).

The survey consisted of three sections. The first included the informed consent form, the second collected socio-demographic characteristics and digital-use information (age, gender, area of residence, occupational status, daily personal internet use, and primary online activity), and the third section contained the psychological instruments administered in Romanian-language versions.

#### 2.1.1 Instruments

##### 2.1.1.1 Problematic internet use

Problematic Internet Use (PIU) was assessed using the 20-item Internet Addiction Test (IAT; Young, 1998) in its validated Romanian version (Tudorel et al., 2019). The scale evaluates various behavioral symptoms, including neglect of duties, sleep deprivation, and difficulties controlling online screen time (e.g., “How often do you anticipate when you will go online again?”). Responses are scored on a 6-point Likert scale ranging from 0 (does not apply) to 5 (always). Items are summed to calculate a total score, with higher values reflecting severe levels of PIU. In the present study, the total scale demonstrated excellent internal consistency (*α* = 0.90), which is highly aligned with the Romanian validation study (*α* = 0.86; Tudorel et al., 2019). PIU was distinguished from the amount of time spent online because the IAT primarily assesses impaired control, cognitive preoccupation, and adverse consequences associated with internet use rather than exposure duration alone. Daily personal internet use was therefore included as a separate behavioral indicator.

##### 2.1.1.2 Perceived social support

Perceived social support was measured using the 24-item Social Provisions Scale (SPS-24; Cutrona and Russell, 1987), translated via a standard back-translation procedure. The instrument spans six distinct relational dimensions (attachment, social integration, reassurance of worth, reliable alliance, guidance, and opportunity for nurturance), using items rated on a 4-point Likert scale from 1 (strongly disagree) to 4 (strongly agree). After reversing the negatively phrased items, a total score was computed (range: 24–96), where higher scores indicate greater perceived social support. The total scale yielded a robust reliability in the current sample (*α* = 0.915), consistent with original findings (*α* = 0.915; Cutrona and Russell, 1987) and recent contextual data (*α* = 0.89, Huțanu and Bogdan, 2025; *α* = 0.93, Zeng and Lau, 2026).

##### 2.1.1.3 Work engagement

Work engagement was evaluated via the short version of the Utrecht Work Engagement Scale (UWES-9; Schaufeli et al., 2006) in its validated Romanian adaptation (Vîrgă et al., 2009). The 9-item scale treats work engagement as a positive, fulfilling work-related state capturing vigor, dedication, and absorption (e.g., “My job inspires me”). Items are rated on a 7-point frequency scale from 0 (never) to 6 (always/every day), and the mean score was calculated. The internal consistency of the total scale in this sample was exceptionally high (*α* = 0.94), matching benchmarks from previous research (*α* = 0.89–0.97, Schaufeli et al., 2006; *α* = 0.89, Vîrgă et al., 2009; *α* = 0.96, Heveri et al., 2025).

##### 2.1.1.4 Work addiction

Work addiction was measured with the 10-item Dutch Work Addiction Scale (DUWAS-10; Schaufeli et al., 2009), adapted into Romanian by Butucescu and Uscătescu (2019). This instrument views workaholism as a negative construct rooted in working excessively and working compulsively (e.g., “I feel that there’s something inside me that drives me to work hard”). Responses are provided on a 4-point Likert scale from 1 (almost never) to 4 (almost always). The overall work addiction index represents the mean of all items, with higher scores denoting elevated compulsive work patterns. The total scale displayed good reliability in the current sample (α = 0.88), showing consistency with the literature (*α* = 0.85, Butucescu and Uscătescu, 2019; *α* = 0.89, Ciobanu et al., 2025). Work addiction was operationalized as compulsive and excessive involvement in work rather than as the amount of time spent using digital technologies for occupational purposes. The DUWAS-10 does not directly assess internet use, occupational screen time, or persistent digital connectivity. It was therefore treated as conceptually distinct from both PIU and daily personal internet use.

##### 2.1.1.5 Depressive symptoms

Depressive symptomatology was quantified using the Romanian version of Beck Depression Inventory–Second Edition (BDI-II; Beck et al., 1996; David and Dobrean, 2012). Respondents evaluated 21 symptoms based on their clinical severity over the past two weeks, utilizing a 4-point ordinal scale (0 to 3). Total scores range from 0 to 63, with higher values signifying increased depressive severity. The current sample yielded a high Cronbach’s alpha (α = 0.93), mirroring the reliability indicators from original and regional validation studies (*α* = 0.93, Beck et al., 1996; *α* = 0.90, Kallay, 2022).

##### 2.1.1.6 Trait anxiety

Trait anxiety was assessed via the Form X2 of the State–Trait Anxiety Inventory (STAI-X2; Spielberger et al., 1970; Pitariu and Peleasa, 2007) in its Romanian version. The 20-item instrument maps an individual’s stable predisposition to perceive environments as dangerous and respond with tension or apprehension (e.g., “I feel blue”). Items are scored on a 4-point frequency scale from 1 (almost never) to 4 (almost always) and summed into a total score (range: 20–80). The internal consistency in the present cohort was excellent (*α* = 0.92), matching standard historical and contemporary psychometric validations (*α* = 0.86–0.92, Spielberger et al., 1970; *α* = 0.89, Grigore and Maftei, 2020; *α* = 0.92).

##### 2.1.1.7 Impulsivity

Trait impulsivity was operationalized using the 30-item Barratt Impulsiveness Scale (BIS-11; Patton et al., 1995) translated into Romanian by the authors using a standard back-translation procedure. The scale measures cognitive, motor, and non-planning components of impulsivity using a 4-point Likert scale ranging from 1 (rarely/never) to 4 (almost always). Total scores range from 30 to 120, where higher scores signal elevated levels of impulsivity. In this study, the global BIS-11 scale exhibited an acceptable internal consistency (*α* = 0.78), aligning with Patton et al.’s (1995) reference range (0.79–0.83) and recent cross-cultural testing evaluations (*α* = 0.69–0.83, Lau et al., 2022; *α* = 0.83, Stanford et al., 2009).

#### 2.1.2 Data analysis

Statistical analyses were performed using IBM SPSS Statistics 26.0 and the PROCESS macro (Version 5.0; Hayes, 2022). Prior to analysis, the dataset was screened for missing values, duplicate responses, coding errors, and univariate or multivariate outliers. No cases met the exclusion criteria.

Descriptive statistics (means, standard deviations, minimum and maximum values, skewness, and kurtosis) were calculated for all continuous variables. Pearson correlation coefficients were computed to examine bivariate associations among the study variables.

A hierarchical multiple regression analysis was conducted to examine the incremental contribution of socio-demographic characteristics, internet use, socio-occupational factors, negative affect, and impulsivity to problematic internet use (PIU). The primary multivariable analyses included age and gender as basic demographic covariates. Daily personal internet use was also included to examine whether the associations with PIU remained after accounting for the self-reported amount of time spent online.

For the primary serial indirect-effect analyses, the standardized scores for depressive symptoms and trait anxiety were averaged to form a composite index representing their shared negative-affect component. Standardization was used because the two instruments had different score ranges. The composite was intended to capture their shared dimension of emotional distress rather than to imply that depressive symptoms and trait anxiety were psychometrically identical.

Hypothesis 3 was tested using serial multiple mediation analyses (PROCESS Model 6; Hayes, 2022). Three separate models examined the indirect associations of perceived social support, work engagement, and work addiction with PIU through negative affect and trait impulsivity in the sequence specified by the theoretical model. Indirect effects were estimated using 10,000 bootstrap resamples with bias-corrected 95% confidence intervals (CIs). Indirect effects were considered statistically significant when the confidence interval did not include zero. To determine whether the findings depended on combining depressive symptoms and trait anxiety into a single index, sensitivity analyses were conducted by repeating the three serial models separately with trait anxiety and depressive symptoms as the first mediator. Trait impulsivity remained the second mediator, and the outcome, covariates, bootstrap procedure, and criteria for statistical significance were identical to those used in the primary models. Because all variables were assessed concurrently, the ordering of negative affect before trait impulsivity was theory-driven and did not represent an empirical test of temporal precedence. Accordingly, the estimated indirect effects were interpreted as statistical associations rather than evidence of causal or temporal mechanisms. To examine the robustness of the findings to alternative covariate specifications, sensitivity analyses were conducted using four adjustment sets: (1) no covariates; (2) the primary covariate set comprising age, gender, and daily personal internet use; (3) an expanded set additionally including area of residence, educational attainment, occupational status, and primary online activity; and (4) the expanded set without daily personal internet use. Categorical covariates with more than two categories were represented by dummy-coded indicators. The hierarchical regression analysis was also repeated using the expanded covariate set. Two sets of supplementary sensitivity analyses were conducted. First, the three theory-specified serial models were re-estimated while mutually adjusting for the socio-occupational predictors; in each model, the other two socio-occupational variables were included as covariates alongside age, gender, and daily personal internet use. Second, the same mutually adjusted models were re-estimated with the mediator order reversed, with trait impulsivity specified as the first mediator and negative affect as the second mediator. Holding the covariate specification constant across the two sets allowed the pattern of indirect associations to be examined under both the theory-specified and alternative mediator orders. These analyses were interpreted as tests of alternative statistical specifications rather than as evidence of temporal ordering. Statistical significance was set at *α* = 0.05.

## 3 Results

Distributional assumptions were evaluated using skewness and kurtosis. As shown in Table 1, all variables met conventional criteria for approximate normality (Kline, 2016), supporting the use of parametric analyses. Descriptive statistics are presented in Table 1.

**Table 1:** Descriptive statistics for the study variables.

Variable	*N*	Mean (M)	Standard deviation (SD)	Skewness	Kurtosis
AGE	215	37.11	9.16	0.36	0.22
PSS	215	85.26	9.84	−1.33	1.68
WE	215	4.09	1.30	−0.52	−0.31
WA	215	2.31	0.70	0.36	−0.41
DS	215	8.10	8.83	1.66	2.96
TA	215	40.33	11.07	0.70	0.24
IMP	215	63.61	11.35	0.21	−0.34
PIU	215	25.33	14.80	0.76	0.36

AGE, age; PSS, perceived social support; WE, work engagement; WA, work addiction; DS, depressive symptoms; TA, trait anxiety; IMP, impulsivity; PIU, problematic internet use.

### 3.1 Bivariate associations

The bivariate correlations were consistent with Hypothesis 1 (see Table 2). PIU was negatively associated with perceived social support and work engagement and positively associated with work addiction, depressive symptoms, trait anxiety, and trait impulsivity. Age was negatively associated with PIU, whereas daily personal internet use was positively associated with it. Depressive symptoms and trait anxiety were strongly correlated (*r* = 0.81, *p* < 0.01), supporting their use as indicators of a shared negative-affect component in the primary serial models. Both were positively associated with impulsivity and showed the expected associations with the socio-occupational variables; however, work addiction was not significantly associated with impulsivity. The remaining correlations, descriptive statistics, and exact effect sizes are reported in Table 2.

**Table 2:** Pearson correlations among the study variables.

Variable	1	2	3	4	5	6	7	8
AGE	-							
PTI	−0.27**	-						
PSS	0.03	−0.04	-					
WE	0.17*	−0.24**	0.36**	-				
WA	−0.02	−0.15*	−0.16*	−0.02	-			
DS	−0.12	0.09	−0.40**	−0.48**	0.30**	-		
TA	−0.19**	0.15*	−0.44**	−0.56**	0.32**	0.81**	-	
IMP	−0.15*	0.21**	−0.30**	−0.37**	0.11	0.44**	0.55**	-
PIU	−0.32**	0.42**	−0.14*	−0.35**	0.23**	0.40**	0.48**	0.47**

AGE, age; PTI, personal time on internet (hours/day); PSS, perceived social support; WE, work engagement; WA, work addiction; DS, depressive symptoms; TA, trait anxiety; IMP, impulsivity; PIU, problematic internet use. * *p* < 0.05; ** *p* < 0.01.

### 3.2 Hierarchical regression analysis

The five-step hierarchical regression analysis was consistent with Hypothesis 2, with each successive block explaining a significant additional proportion of variance in PIU (see Table 3). The final model explained 45.5% of the variance in PIU (adjusted R^2^ = 0.431). In this model, age was negatively associated with PIU, whereas daily personal internet use, work addiction, and trait impulsivity showed significant positive associations. Gender, perceived social support, work engagement, depressive symptoms, and trait anxiety were not significantly associated with PIU after all variables were considered. Complete results for each step, including regression coefficients, changes in explained variance, and model statistics, are presented in Table 3.

**Table 3:** Hierarchical multiple regression analysis predicting problematic internet use.

Variable	B	SE	*β*	*t*	*p*	ΔR^2^
Step 1:						
AGE	−0.54	0.11	−0.34	−4.91	0.000	
GN	1.72	2.06	0.06	0.84	0.405	0.104***
R^2^ = 0.104, Δ*F*(2, 212) = 12.31						
Step 2: Time on internet						
PTI	5.36	0.95	0.37	5.65	0.000	0.118***
R^2^ = 0.222, Δ*F*(1, 211) = 31.93						
Step 3:						
PSS	−0.00	0.09	−0.00	−0.02	0.982	
WE	−2.63	0.71	−0.23	−3.71	0.000	
WA	5.89	1.23	0.28	4.78	0.000	0.131***
R^2^ = 0.352, Δ*F*(3, 208) = 13.98						
Step 4:						
DS	0.09	0.15	0.06	0.60	0.549	
TA	0.41	0.14	0.31	3.01	0.003	0.061***
R^2^ = 0.414, Δ*F*(2, 206) = 10.80						
Step 5:						
AGE	−0.27	0.09	−0.17	−2.83	0.005	
GN	1.89	1.82	0.06	1.04	0.299	
PTI	4.25	0.85	0.30	5.01	0.000	
PSS	0.18	0.09	0.12	1.94	0.054	
WE	−0.77	0.75	−0.07	−1.03	0.305	
WA	4.21	1.21	0.20	3.49	0.001	
DS	0.10	0.15	0.06	0.68	0.495	
TA	0.24	0.14	0.18	1.78	0.077	
IMP	0.32	0.08	0.25	3.93	0.000	0.041***
Final model: R^2^ = 0.455, Δ*F*(1, 205) = 15.40, R^2^adj = 0.431						

AGE, age; GN, Gender; PTI, personal time on internet (hours/day); PSS, perceived social support; WE, work engagement; WA, work addiction; DS, depressive symptoms; TA, trait anxiety; IMP, impulsivity. Tolerance = 0.25–0.82; VIF = 1.22–3.97 and Durbin-Watson (DW = 1.943). *** *p* < 0.001.

### 3.3 Theory-specified serial indirect effect analyses

To evaluate Hypothesis 3, theory-specified serial indirect-effect analyses were conducted using PROCESS Model 6 (Hayes, 2022). Three primary models separately examined the direct and indirect associations of perceived social support (Model 1), work engagement (Model 2), and work addiction (Model 3) with problematic internet use (PIU), with negative affect specified as the first mediator and trait impulsivity as the second mediator. The primary adjustment set comprised age, gender, and daily personal internet use. Because all variables were measured concurrently, the ordering of negative affect before trait impulsivity represents an analytical specification derived from the theoretical model and should not be interpreted as evidence of temporal precedence or causal direction (Figure 1).

**Figure 1:**
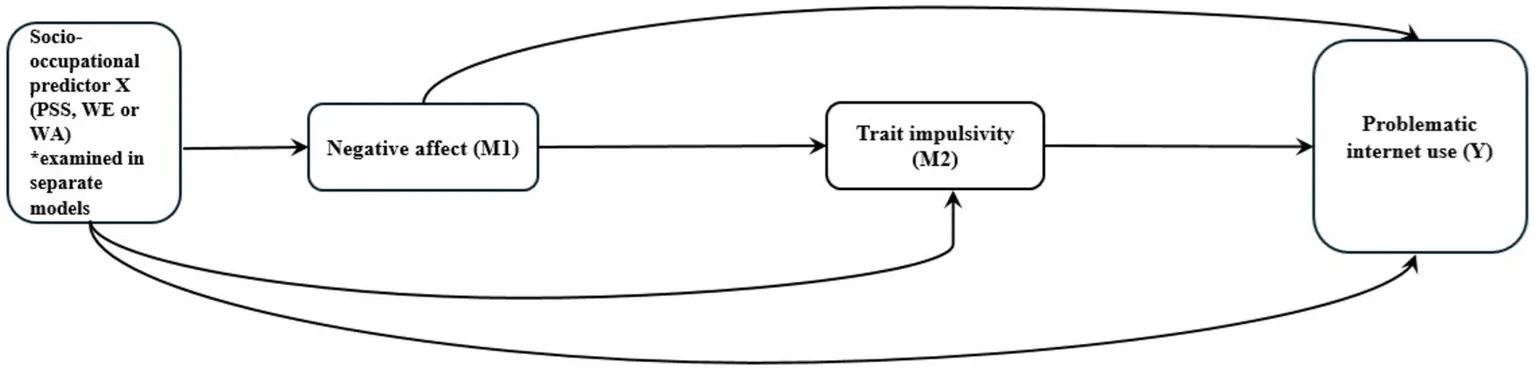
Theory-specified serial indirect-effect model. Perceived social support, work engagement, and work addiction were examined separately as the socio-occupational predictor (X). Negative affect was specified as the first mediator (M₁), trait impulsivity as the second mediator (M₂), and problematic internet use as the outcome (Y). Arrows represent regression associations estimated within the statistical models and do not indicate established temporal or causal relationships. Primary models were adjusted for age, gender, and daily personal internet use.

The findings were partially consistent with Hypothesis 3. As hypothesized, significant indirect associations through negative affect were observed for perceived social support and work engagement, and the hypothesized serial indirect association through negative affect and trait impulsivity was observed for work addiction. However, the same serial indirect association was also significant for perceived social support and work engagement, extending beyond the predictor-specific pattern originally hypothesized. Across all three models, the indirect associations through trait impulsivity alone were not statistically significant; however, these findings should not be interpreted as evidence that such associations are absent. The direct associations of perceived social support and work engagement with PIU were not statistically significant after negative affect and impulsivity were included in the models, whereas work addiction retained a significant positive direct association with PIU. Exact coefficients, significance levels, and bootstrap confidence intervals are presented in Table 4.

**Table 4:** Direct, indirect, and total associations in the theory-specified serial models with the primary covariate set.

Effect	Model 1 B (95% CI)	Model 2 B (95% CI)	Model 3 B (95% CI)
X → Negative affect	−0.05*** (−0.06, −0.04)	−0.40*** (−0.48, −0.31)	0.46*** (0.29, 0.63)
X → Impulsivity	−0.13 (−0.29, 0.02)	−0.80 (−2.03, 0.43)	−0.62 (−2.64, 1.40)
Negative affect → Impulsivity	5.20***	5.29***	6.03***
Negative affect → PIU	5.00***	4.19***	3.30**
Impulsivity → PIU	0.33***	0.31***	0.32***
Direct effect (c′)	0.15	−0.25	3.96**
Total effect (c)	−0.21*	−2.82***	6.17***
Indirect via negative affect	−0.23 (−0.37, −0.12)	−1.67 (−2.74, −0.70)	1.52 (0.46, 2.75)
Indirect via impulsivity	−0.04 (−0.12, 0.01)	−0.25 (−0.73, 0.14)	−0.20 (−0.90, 0.61)
Serial indirect effect	−0.08 (−0.14, -0.03)	−0.65 (−1.24, −0.22)	0.89 (0.33, 1.63)
Total indirect effect	−0.36 (−0.52, −0.22)	−2.57 (−3.71, −1.54)	2.21 (0.92, 3.65)

Unstandardized regression coefficients (B) are reported. Values in parentheses represent 95% percentile bootstrap confidence intervals (10,000 resamples). All models controlled for age, gender, daily personal internet use. The indirect effects represent (1) the pathway through negative affect, (2) the pathway through trait impulsivity, and (3) the serial pathway through negative affect and trait impulsivity. PIU, problematic internet use. *p < 0.05, **p < 0.01, ***p < 0.001.

### 3.4 Sensitivity analyses for covariate specification

The hierarchical regression analysis was repeated using an expanded adjustment set that additionally included area of residence, educational attainment, occupational status, and primary online activity (see Supplementary Table S1). The additional covariate block explained 7.3% of the variance in PIU (*p* = 0.005), and the final model accounted for 48.7% of the variance. The overall pattern for the primary study variables remained comparable to that obtained in the original analysis: age was negatively associated with PIU, whereas daily personal internet use, work addiction, and trait impulsivity showed significant positive associations. One educational indicator was also significant in the final model, although this estimate was based on a small subgroup and should be interpreted cautiously.

The serial indirect-effect models were subsequently compared across four covariate specifications: no covariates, the primary covariate set, the expanded covariate set, and the expanded set without daily personal internet use (see Supplementary Table S2). Across all specifications and all three socio-occupational predictors, the indirect associations through negative affect alone and through the theory-specified sequence involving negative affect and trait impulsivity remained significant. The indirect association through trait impulsivity alone remained non-significant. However, some direct and total associations varied across adjustment sets. In particular, the direct and total associations involving perceived social support were sensitive to covariate specification, and the direct association between work addiction and PIU was not significant in the unadjusted model but was significant in the adjusted models. Thus, the principal indirect-association pattern was robust across covariate specifications, whereas some direct and total associations were less stable.

### 3.5 Sensitivity analyses for the operationalization of negative affect

The three serial indirect-effect models were repeated separately using trait anxiety and depressive symptoms as the first mediator. As shown in Supplementary Tables S3, S4, the models using trait anxiety reproduced the pattern obtained with the negative affect composite across all three socio-occupational variables. The models using depressive symptoms also retained significant indirect effects through depressive symptoms, significant serial indirect effects through depressive symptoms and impulsivity, and the same pattern of direct effects, although the indirect effects were generally smaller. In addition, significant indirect effects through impulsivity alone emerged for perceived social support and work engagement in the depression models. Across all nine models, the total indirect effect and the theory-specified serial indirect effect remained significant, while the direct effect remained significant only for work addiction. Thus, the principal findings did not depend solely on combining depression and anxiety, although the sensitivity analyses also indicated that these constructs were not interchangeable.

### 3.6 Sensitivity analyses for mutual adjustment and alternative mediator ordering

Finally, the theory-specified models were re-estimated while mutually adjusting for the socio-occupational predictors, and the same mutually adjusted models were subsequently estimated with the mediator order reversed (see Supplementary Table S5). Under mutual adjustment, the theory-specified serial indirect association through negative affect and trait impulsivity remained significant for all three predictors, whereas the indirect association through impulsivity alone remained non-significant. The total association of perceived social support with PIU was no longer significant, while work addiction retained a significant direct association with PIU. When the mediator order was reversed, the serial indirect association through trait impulsivity and negative affect was significant for perceived social support and work engagement, but not for work addiction. In the reversed models, the indirect association through impulsivity alone was significant for perceived social support and work engagement, whereas only the specific indirect association through negative affect was significant for work addiction. Total indirect associations remained significant across all models.

## 4 Discussion

The present study examined the relationships between socio-occupational factors, negative affect, impulsivity, and problematic internet use (PIU) in a sample of Romanian adults. Overall, the findings were consistent with several components of the proposed theoretical model. Perceived social support and work engagement showed significant indirect associations with PIU through negative affect and impulsivity, whereas work addiction showed significant direct and indirect associations with PIU. These findings suggest that psychological and occupational factors are associated with PIU after accounting for demographic characteristics and time spent online.

The observed pattern is consistent with the Interaction of Person–Affect–Cognition–Execution (I-PACE) model (Brand et al., 2025), which proposes that distal vulnerabilities and resources influence problematic internet use through proximal affective and cognitive mechanisms. Within the present model, socio-occupational factors were conceptualized as distal contextual variables, negative affect was conceptualized as a proximal emotional factor, and impulsivity represented a self-regulatory factor associated with PIU. However, these theoretical roles and their proposed temporal ordering cannot be established from the present cross-sectional data.

The findings related to Hypotheses 1 and 2 were generally consistent with previous research. PIU was negatively associated with perceived social support and work engagement and positively associated with work addiction, depressive symptoms, trait anxiety, and impulsivity, in line with recent cross-cultural studies (Augner et al., 2023; Cai et al., 2023; Gu et al., 2024). Furthermore, hierarchical regression analyses showed that socio-occupational variables, negative affect, and impulsivity explained additional variance in PIU beyond demographic characteristics and daily internet use. These findings indicate that, within the present predominantly urban and highly educated sample, PIU was associated not only with behavioral factors such as screen time, but also with emotional and occupational characteristics.

A key finding of the present study concerns the indirect associations involving negative affect. Across all three indirect-effect models, significant indirect effects through negative affect were observed in the associations between the socio-occupational factors and PIU. This pattern is consistent with the Compensatory Internet Use Theory (Kardefelt-Winther, 2014), which proposes that problematic internet use may represent a maladaptive strategy for regulating negative emotional states rather than a direct consequence of technology use itself. One possible interpretation is that individuals reporting higher levels of depression or anxiety may also be more likely to rely on online environments to manage psychological distress, which could be associated with more maladaptive patterns of internet use. However, the reverse direction, whereby problematic internet use contributes to emotional distress, remains equally plausible. The sensitivity analyses further clarified this pattern. Trait anxiety closely reproduced the findings obtained with the composite index, whereas depressive symptoms yielded the same general pattern of significant mediator-specific and serial indirect effects, although the estimates were generally smaller and additional significant indirect effects through impulsivity alone emerged for social support and work engagement. These findings support the robustness of the overall indirect-effect pattern while also indicating that depression and trait anxiety should not be treated as interchangeable indicators of emotional distress.

For perceived social support, the direct association with PIU became non-significant after accounting for the mediators, indicating that the association between social support and PIU was statistically accounted for primarily by the proposed emotional pathway in the present model. This finding is consistent with the stress-buffering hypothesis, according to which supportive interpersonal relationships may be associated with lower psychological distress and a reduced tendency to seek emotional compensation through online activities (Jia et al., 2022; Meshi and Ellithorpe, 2021). Nevertheless, the present findings do not establish that social support temporally precedes or reduces emotional distress or problematic internet use.

A similar pattern was observed for work engagement. Although work engagement was initially associated with lower PIU, its direct association with PIU was no longer significant after negative affect and impulsivity were included in the model. Individuals who experience higher levels of engagement may also experience lower emotional distress, which could be associated with a lower likelihood of reporting online activities as a coping strategy. This interpretation is consistent with previous research describing work engagement as a positive occupational resource associated with psychological well-being and adaptive functioning (Schaufeli et al., 2008; Shimazu et al., 2012). However, alternative directions of association cannot be excluded based on the present cross-sectional design.

An additional contribution of the present study concerns the role of impulsivity. The findings were only partially consistent with the predictor-specific pattern proposed in Hypothesis 3. Although the serial indirect association through negative affect and trait impulsivity was specifically hypothesized for work addiction, statistically significant serial indirect associations were also observed for perceived social support and work engagement. These additional findings therefore extended beyond the original hypothesis and should be interpreted cautiously pending replication. Although impulsivity was associated with PIU and explained additional variance in the regression analysis, the indirect association through impulsivity alone was not statistically significant in any of the three models. This finding indicates that these estimates were not statistically distinguishable from zero in the present sample and should not be interpreted as evidence that such associations are absent in the population. Instead, significant indirect associations involving impulsivity were observed within the theory-specified sequence that also included negative affect. This pattern is compatible with the possibility that the association between socio-occupational factors and impulsivity may be linked to negative affect within the tested statistical models. This interpretation is consistent with models of self-regulation suggesting that elevated emotional distress may be associated with reduced cognitive control and greater impulsive responding (Augner et al., 2023). However, given the cross-sectional nature of the present study, the direction and temporal ordering of the associations among negative affect, impulsivity, and PIU cannot be determined. Alternative sequences, including impulsivity preceding negative affect or PIU contributing to both negative affect and impulsivity, therefore remain plausible and require examination using longitudinal designs.

The mutually adjusted analyses showed that the theory-specified serial indirect association remained significant for all three socio-occupational predictors, although the total association of perceived social support with PIU was no longer significant. When the mediator order was reversed under the same covariate specification, the serial indirect association was significant for perceived social support and work engagement, but not for work addiction. Thus, both mediator orders were compatible with the findings for perceived social support and work engagement, whereas work addiction was more consistent with the theory-specified ordering. Because the PIU regression equation contained the same variables under both mediator orderings, reversing the mediators changed the decomposition of the total indirect association rather than the outcome equation itself. Accordingly, these cross-sectional models cannot establish temporal precedence or determine which ordering better represents the underlying process.

Work addiction showed a different pattern from the other socio-occupational variables. In contrast to perceived social support and work engagement, work addiction retained a significant direct association with PIU after negative affect and impulsivity were included in the model, while also showing significant indirect associations through these variables. Work addiction and PIU were operationalized using measures targeting conceptually distinct domains: compulsive and excessive involvement in work and impaired control over internet use, respectively. Moreover, their relatively modest bivariate association (*r* = 0.23) does not suggest construct equivalence, although it does not constitute a formal test of discriminant validity. One possible explanation for the observed association is that compulsive work involvement may coexist with persistent occupational demands requiring continuous digital connectivity. Individuals with high levels of work addiction often experience difficulty disengaging from work, remain connected to work-related communication outside working hours, and may rely heavily on digital technologies to maintain this continuous involvement (Andreassen, 2014; Schaufeli et al., 2009). However, occupational digital engagement was not directly measured in the present study, and this interpretation therefore remains speculative. The observed association may also partly reflect conceptual or behavioral overlap between compulsive work involvement, extensive digital connectivity, and some manifestations of PIU, as well as variance attributable to the shared self-report measurement method. These possibilities warrant further investigation using measures that distinguish occupational from non-occupational internet use and latent or multimethod approaches designed to evaluate the discriminant validity of the relevant constructs.

Overall, the present findings extend previous research by integrating social, occupational, emotional, and self-regulatory factors within a single statistical model of adult PIU. In particular, the results identified negative affect as a prominent indirect pathway in the tested models, while suggesting that impulsivity was involved primarily in the serial indirect association that also included negative affect. These findings are broadly consistent with theoretically informed models of PIU and may guide longitudinal research designed to test the direction and temporal ordering of the observed associations.

### 4.1 Practical implications

Beyond their theoretical contribution, these findings may generate hypotheses for future workplace prevention and intervention research concerning problematic internet use among adults. Because negative affect was involved in significant indirect associations between the socio-occupational variables and PIU, future studies could evaluate whether interventions targeting emotion regulation—such as stress-management or mindfulness-based programs—offer benefits when implemented alongside, or compared with, approaches focused on limiting screen time. Organizations could also evaluate policies that support psychological detachment from work (e.g., clearer boundaries around after-hours connectivity), particularly for employees showing signs of work addiction, as well as initiatives that strengthen perceived social support in the workplace. However, the present cross-sectional findings do not demonstrate that these approaches would reduce PIU or that they would be more effective than interventions focused on internet-use behavior. These possibilities should therefore be tested in longitudinal, experimental, or intervention studies before specific practical recommendations are made.

### 4.2 Limitations

Several limitations should be considered when interpreting the present findings. First, the cross-sectional design precludes conclusions regarding temporal ordering or causal relationships among the study variables. Although the proposed serial indirect effect model was theoretically informed by the I-PACE framework, the data cannot establish that socio-occupational factors precede negative affect, that negative affect precedes impulsivity, or that impulsivity subsequently contributes to PIU. Alternative sequences, including PIU contributing to negative affect or impulsivity influencing negative affect, remain equally plausible. Longitudinal and ecological momentary assessment (EMA) studies are needed to examine the direction and temporal ordering of these associations over time.

Second, participants were recruited through convenience sampling via social and professional networking platforms, resulting in a highly selected sample. Approximately 91% of participants lived in urban areas, and more than half held postgraduate or doctoral qualifications. The sample therefore differs substantially from the broader Romanian adult population in terms of residential background and educational attainment. Furthermore, because bachelor’s and post-secondary non-tertiary qualifications were combined into a single response category, their respective proportions could not be determined retrospectively. This limited the precision of comparisons between the educational composition of the sample and national educational-attainment statistics. Moreover, recruiting active internet users through online platforms may have preferentially attracted individuals with greater digital access, familiarity, or engagement, potentially introducing selection bias in a study focused on problematic internet use. Consequently, the findings should not be generalized to Romanian adults as a whole, particularly those living in rural areas, those with lower educational attainment, or individuals with more limited digital access. Future research should use more diverse recruitment channels and sampling strategies to examine whether the observed associations replicate across more heterogeneous segments of the Romanian population.

Third, all variables were assessed using self-report measures, increasing the possibility of common-method variance and response biases such as social desirability. The use of a single assessment method may have inflated some of the observed associations and does not permit common-method variance to be fully distinguished from substantive relationships among the constructs. Future studies may benefit from combining self-report instruments with behavioral or objective indicators of internet use.

Fourth, daily internet use was assessed using categorical self-reported estimates rather than objective digital tracking data. More precise measures of online behavior may improve the accuracy of future research. In addition, the study did not distinguish sufficiently between occupational and non-occupational digital engagement, limiting our ability to determine whether the association between work addiction and PIU partly reflected persistent work-related connectivity. Moreover, participants who were outside the active workforce completed the work engagement and work addiction measures retrospectively with reference to their most recent job. These ratings may have been affected by recall bias and may not fully represent the participants’ current occupational context.

Fifth, the primary adjustment set was not derived from a prespecified theoretical framework for covariate selection and did not include all measured socio-demographic and digital-use characteristics. Sensitivity analyses using alternative specifications showed that the indirect associations through negative affect and through the theory-specified sequence involving negative affect and trait impulsivity remained consistent across adjustment sets, including models with no covariates and models without daily personal internet use. However, some direct and total associations, particularly those involving perceived social support and work addiction, varied according to the covariate specification. These estimates should therefore be interpreted cautiously. Although the expanded models additionally included educational attainment, occupational status, area of residence, and primary online activity, other unmeasured characteristics may still have affected the observed associations. Future studies should specify covariates *a priori* on the basis of theory and previous evidence.

Sixth, the *a priori* power analysis was designed for the hierarchical regression analysis rather than specifically for the serial indirect effects that represented a central component of the study. Although bootstrapped confidence intervals were used to estimate the indirect effects, the sample may have provided limited precision for detecting and estimating more complex serial indirect effects. Future studies should use sample-size planning procedures specifically designed for indirect-effect models.

Seventh, some conceptual and behavioral overlap may exist among PIU, work addiction, daily internet use, and occupational digital engagement. Although work addiction and PIU were assessed using instruments targeting distinct constructs and showed a relatively modest correlation (*r* = 0.23), the present study did not formally test their discriminant validity. Moreover, occupational digital engagement was not measured directly. Therefore, the observed association may partly reflect overlapping manifestations of excessive or poorly regulated behavior or shared self-report method variance. Future research should distinguish occupational from personal internet use and evaluate discriminant validity using latent measurement models and multimethod data.

Eighth, formal Romanian validation evidence was not available for the Romanian-language versions of the SPS-24 and BIS-11 used in the present study. Although both instruments were translated using a back-translation procedure and demonstrated acceptable to excellent internal consistency in the present sample, translation procedures and internal consistency alone do not establish construct validity or measurement equivalence. Consequently, findings involving perceived social support and trait impulsivity should be interpreted with appropriate caution. Future research should formally evaluate the psychometric properties of these Romanian-language versions.

Finally, although the negative affect composite was theoretically grounded in the shared transdiagnostic component of depression and anxiety, the two constructs were measured using distinct instruments and should not be regarded as psychometrically identical. The sensitivity analyses indicated that the principal indirect-effect pattern was retained when depressive symptoms and trait anxiety were examined separately, although some differences also emerged. Moreover, the present study used observed scores rather than a latent negative-affect construct. Future studies with larger samples could evaluate the shared and construct-specific components of depression and anxiety using latent or bifactor measurement models.

Despite these limitations, the present study contributes to previous research by integrating social, occupational, emotional, and self-regulatory factors within a single theory-informed statistical model of problematic internet use. Future longitudinal and ecological momentary studies are needed to replicate these findings and further examine the direction and temporal ordering of the associations among socio-occupational factors, negative affect, impulsivity, and PIU.

### 4.3 Future research directions

The present findings suggest several directions for future research. First, longitudinal and ecological momentary assessment (EMA) studies are needed to clarify the temporal ordering proposed by the present serial mediation model and to determine whether changes in negative affect precede increases in impulsivity and subsequent problematic internet use over time.

Second, future research should distinguish between different forms of problematic internet use. The present study focused on PIU as a general construct; however, the mechanisms identified here may differ across specific online behaviors such as social media use, online gaming, video streaming, or work-related digital overuse.

Third, intervention studies may examine whether reducing negative affect through psychological interventions (e.g., cognitive-behavioral therapy, mindfulness-based interventions, or emotion regulation training) leads to subsequent reductions in impulsive responding and problematic internet use. Such studies would provide a stronger test of the mechanisms proposed by the present model.

Finally, future studies should further investigate occupational characteristics associated with problematic internet use. Given the direct association observed for work addiction, it would be valuable to examine additional work-related variables, such as boundary management, remote work demands, technostress, organizational expectations of constant availability, and work–life balance.

## 5 Conclusion

The present study examined the relationships between socio-occupational factors, negative affect, impulsivity, and problematic internet use (PIU) in a predominantly urban and highly educated sample of Romanian adults. Overall, the findings were consistent with several components of the proposed theoretical model. Perceived social support and work engagement showed significant indirect associations with PIU through negative affect and impulsivity, whereas work addiction showed significant direct and indirect associations with PIU.

Among the examined variables, negative affect was involved in a prominent indirect pathway linking socio-occupational characteristics to PIU. The theory-specified serial indirect associations remained significant for all three predictors after mutual adjustment, whereas the indirect associations through impulsivity alone were not significant. When the mediator order was reversed, the serial indirect association was significant for perceived social support and work engagement, but not for work addiction. These cross-sectional findings do not establish temporal ordering, and alternative directions remain plausible. Within this predominantly urban and highly educated Romanian adult sample, PIU was associated with emotional and occupational characteristics beyond the amount of time spent online.

From a practical perspective, the findings highlight emotion regulation, social support, work engagement, and work addiction as variables that warrant examination in future prevention and intervention research. However, the present study did not test whether modifying these factors reduces PIU or whether emotion-focused interventions are more effective than approaches focused on internet-use behavior. Longitudinal, experimental, and ecological momentary studies are needed to examine the temporal ordering of the observed associations and to evaluate these potential intervention targets. Overall, the present study contributes an integrated account of the associations among social, occupational, emotional, and self-regulatory factors in relation to problematic internet use.

## Data Availability

The raw data supporting the conclusions of this article will be made available by the authors, without undue reservation.
