# Supplementary material for: Socio-occupational factors and problematic internet use: indirect associations involving negative affect and impulsivity in a predominantly urban, highly educated Romanian adult sample
Source: Front Psychol. 2026 Oct 30;17:1940887. doi: 10.3389/fpsyg.2026.1940887 (PMC13759804; doi:10.3389/fpsyg.2026.1940887)
Supplement: Supplementary file 1 [file Supplementary_file_1.docx]

Supplementary Material

**Supplementary Table 1. Hierarchical Multiple Regression Analysis Predicting Problematic Internet Use With the Four Additional Covariates**

| Variable | *B* | *SE* | β | *t* | *p* | ΔR² |
| --- | --- | --- | --- | --- | --- | --- |
| Step 1: | | | | | | |
| AGE | -.54 | .11 | -.34 | -4.91 | < .001 |  |
| GN | 1.72 | 2.06 | .06 | .84 | .405 | .104*** |
| R² = .104, Δ*F*(2, 212) = 12.31 | | | | | | |
| Step 2: Time on internet | | | | | | |
| PTI | 5.36 | .95 | .37 | 5.65 | < .001 | .118*** |
| R² = .222, Δ*F*(1, 211) = 31.93 | | | | | | |
| Step 3: Four additional covariates | | | | | | |
| *Area of residence (ref.: urban)* | | | | | | |
| Rural | -3.50 | 3.11 | -.07 | -1.12 | .263 |  |
| *Educational attainment (ref.: bachelor’s or post-secondary degree)* | | | | | | |
| High school or vocational school | -9.94 | 3.98 | -.16 | -2.50 | .013 |  |
| Postgraduate or doctoral degree | 1.16 | 1.94 | .04 | .60 | .550 |  |
| *Occupational status (ref.: employed)* | | | | | | |
| Self-employed or freelancer | -2.84 | 3.19 | -.06 | -.89 | .374 |  |
| Outside the active workforce | 6.98 | 3.06 | .14 | 2.28 | .024 |  |
| *Primary online activity (ref.: social communication)* | | | | | | |
| Entertainment | .27 | 2.03 | .01 | .13 | .896 |  |
| Utilitarian or informational | -5.17 | 2.92 | -.11 | -1.77 | .078 | .073** |
| R² = .295, Δ*F*(7, 204) = 3.03 | | | | | | |
| Step 4: | | | | | | |
| PSS | .01 | .09 | .01 | .08 | .936 |  |
| WE | -2.31 | .72 | -.20 | -3.19 | .002 |  |
| WA | 5.51 | 1.23 | .26 | 4.47 | < .001 | .103*** |
| R² = .398, Δ*F*(3, 201) = 11.42 | | | | | | |
| Step 5: | | | | | | |
| DS | .14 | .15 | .08 | .91 | .365 |  |
| TA | .33 | .14 | .24 | 2.31 | .022 | .047*** |
| R² = .445, Δ*F*(2, 199) = 8.35 | | | | | | |
| Step 6: | | | | | | |
| AGE | -.25 | .10 | -.16 | -2.51 | .013 |  |
| GN | 2.56 | 1.97 | .09 | 1.30 | .194 |  |
| PTI | 4.30 | .87 | .30 | 4.92 | < .001 |  |
| *Area of residence (ref.: urban)* | | | | | | |
| Rural | -2.50 | 2.75 | -.05 | -.91 | .365 |  |
| *Educational attainment (ref.: bachelor’s or post-secondary degree)* | | | | | | |
| High school or vocational school | -7.75 | 3.51 | -.12 | -2.21 | .028 |  |
| Postgraduate or doctoral degree | .45 | 1.71 | .02 | .26 | .794 |  |
| *Occupational status (ref.: employed)* | | | | | | |
| Self-employed or freelancer | -2.23 | 2.81 | -.04 | -.79 | .429 |  |
| Outside the active workforce | 4.65 | 2.72 | .10 | 1.71 | .089 |  |
| *Primary online activity (ref.: social communication)* | | | | | | |
| Entertainment | -.63 | 1.78 | -.02 | -.35 | .725 |  |
| Utilitarian or informational | -3.20 | 2.58 | -.07 | -1.24 | .216 |  |
| PSS | .17 | .09 | .11 | 1.80 | .073 |  |
| WE | -.69 | .77 | -.06 | -.91 | .366 |  |
| WA | 3.97 | 1.22 | .19 | 3.25 | .001 |  |
| DS | .15 | .15 | .09 | 1.01 | .312 |  |
| TA | .15 | .14 | .12 | 1.08 | .283 |  |
| IMP | .33 | .08 | .25 | 4.04 | < .001 | .042*** |
| Final model: R² = .487, Δ*F*(1, 198) = 16.34, R²adj = .445 | | | | | | |

*Note 1. AGE = age; GN = gender; PTI = personal time on internet (hours/day); PSS = perceived social support; WE = work engagement; WA = work addiction; DS = depressive symptoms; TA = trait anxiety; IMP = impulsivity; ref. = reference category. The four additional covariates (area of residence, educational attainment, occupational status, and primary online activity) were entered in one block as seven dummy-coded indicators (0/1); each coefficient compares the indicated category with the reference category, and the reference category for educational attainment also includes “other” (n = 2). Note 2. Tolerance = .23–.89; VIF = 1.13–4.38 and Durbin-Watson (DW = 2.007). Note 3. **p < .01, ***p < .001.*

**Supplementary Table 2. Direct, Indirect, and Total Associations Under Alternative Covariate Specifications**

| **Effect** | **Model 1 B (95% CI)** | **Model 2 B (95% CI)** | **Model 3 B (95% CI)** |
| --- | --- | --- | --- |
| *No covariates* | | | |
| X → Negative affect | -.04*** (-.05,-.03) | -.40*** (-.49,-.32) | .44*** (.27,.62) |
| X → Impulsivity | -.10 (-.25,.05) | -1.03 (-2.24,.17) | -1.03 (-3.02,.95) |
| Negative affect → Impulsivity | 5.77*** | 5.44*** | 6.47*** |
| Negative affect → PIU | 5.38*** | 3.96*** | 4.09*** |
| Impulsivity → PIU | .42*** | .39*** | .42*** |
| Direct effect (c′) | .16 | -1.16 | 2.39 |
| Total effect (c) | -.21* | -4.01*** | 4.97*** |
| Indirect via negative affect | -.23 (-.37,-.11) | -1.60 (-2.83,-.51) | 1.82 (.62,3.27) |
| Indirect via impulsivity | -.04 (-.13,.03) | -.40 (-1.00,.05) | -.43 (-1.37,.49) |
| Serial indirect effect | -.10 (-.18,-.04) | -.85 (-1.52,-.32) | 1.19 (.44,2.15) |
| Total indirect effect | -.37 (-.55,-.23) | -2.85 (-4.04,-1.79) | 2.59 (.96,4.28) |
| *Expanded covariate set* | | | |
| X → Negative affect | -.05*** (-.06,-.03) | -.39*** (-.48,-.31) | .47*** (.30,.64) |
| X → Impulsivity | -.12 (-.28,.03) | -.73 (-2.00,.54) | -.51 (-2.59,1.58) |
| Negative affect → Impulsivity | 5.16*** | 5.26*** | 5.91*** |
| Negative affect → PIU | 4.43*** | 3.69*** | 2.78** |
| Impulsivity → PIU | .33*** | .31*** | .32*** |
| Direct effect (c′) | .14 | -.17 | 3.74** |
| Total effect (c) | -.18 | -2.50*** | 5.78*** |
| Indirect via negative affect | -.20 (-.33,-.08) | -1.45 (-2.53,-.48) | 1.31 (.23,2.58) |
| Indirect via impulsivity | -.04 (-.11,.02) | -.23 (-.71,.16) | -.16 (-.86,.67) |
| Serial indirect effect | -.08 (-.14,-.03) | -.65 (-1.20,-.23) | .90 (.34,1.60) |
| Total indirect effect | -.32 (-.47,-.18) | -2.33 (-3.45,-1.34) | 2.04 (.73,3.52) |
| *Expanded covariate set without daily personal internet use* | | | |
| X → Negative affect | -.04*** (-.06,-.03) | -.39*** (-.48,-.31) | .44*** (.27,.62) |
| X → Impulsivity | -.10 (-.26,.06) | -.95 (-2.22,.32) | -.96 (-3.03,1.11) |
| Negative affect → Impulsivity | 5.50*** | 5.29*** | 6.21*** |
| Negative affect → PIU | 4.75*** | 3.44** | 3.17** |
| Impulsivity → PIU | .40*** | .37*** | .39*** |
| Direct effect (c′) | .20* | -.67 | 2.59* |
| Total effect (c) | -.15 | -3.16*** | 4.69*** |
| Indirect via negative affect | -.21 (-.35,-.09) | -1.36 (-2.51,-.34) | 1.40 (.31,2.67) |
| Indirect via impulsivity | -.04 (-.12,.03) | -.36 (-.90,.10) | -.38 (-1.28,.53) |
| Serial indirect effect | -.10 (-.16,-.04) | -.78 (-1.35,-.34) | 1.08 (.43,1.86) |
| Total indirect effect | -.35 (-.51,-.20) | -2.49 (-3.68,-1.44) | 2.10 (.60,3.68) |

*Results for the primary covariate set, comprising age, gender, and daily personal internet use, are reported in Table 4. The expanded covariate set additionally included area of residence, educational attainment, occupational status, and primary online activity.*

**Supplementary Table 3. Direct, Indirect, and Total Associations of the Serial Multiple Mediation Models With Trait Anxiety Predicting Problematic Internet Use**

| **Effect** | **Model 1 B (95% CI)** | **Model 2 B (95% CI)** | **Model 3 B (95% CI)** |
| --- | --- | --- | --- |
| X → Trait anxiety | -.54*** (-.67,-.41) | -4.70*** (-5.67,-3.73) | 5.22*** (3.21,7.23) |
| X → Impulsivity | -.11 (-.26,.04) | -.53 (-1.75,.68) | -.75 (-2.73,1.22) |
| Trait anxiety → Impulsivity | .49*** | .51*** | .56*** |
| Trait anxiety → PIU | .44*** | .37*** | .29** |
| Impulsivity → PIU | .31*** | .30*** | .31*** |
| Direct effect (c′) | .15 | -.21 | 3.97** |
| Total effect (c) | -.21* | -2.82*** | 6.17*** |
| Indirect via trait anxiety | -.24 (-.37,-.13) | -1.75 (-2.83,-.75) | 1.53 (.55,2.67) |
| Indirect via impulsivity | -.03 (-.10,.02) | -.16 (-.60,.22) | -.23 (-.89,.48) |
| Serial indirect effect | -.08 (-.15,-.03) | -.70 (-1.34,-.21) | .90 (.32,1.66) |
| Total indirect effect | -.36 (-.51,-.23) | -2.61 (-3.81,-1.55) | 2.20 (.96,3.59) |

*Note. Unstandardized regression coefficients (B) are reported. Values in parentheses represent 95% percentile bootstrap confidence intervals (10,000 resamples). All models controlled for age, gender, and daily personal internet use. The indirect effects represent (1) the pathway through trait anxiety, (2) the pathway through trait impulsivity, and (3) the serial pathway through trait anxiety and trait impulsivity. PIU = problematic internet use. *p < .05, **p < .01, ***p < .001.*

**Supplementary Table 4. Direct, Indirect, and Total Associations of the Serial Multiple Mediation Models With Depressive Symptoms Predicting Problematic Internet Use**

| **Effect** | **Model 1 B (95% CI)** | **Model 2 B (95% CI)** | **Model 3 B (95% CI)** |
| --- | --- | --- | --- |
| X → Depressive symptoms | -.39*** (-.50,-.28) | -3.28*** (-4.12,-2.45) | 3.94*** (2.29,5.59) |
| X → Impulsivity | -.21** (-.37,-.06) | -1.53* (-2.74,-.31) | .09 (-2.01,2.18) |
| Depressive symptoms → Impulsivity | .42*** | .42*** | .52*** |
| Depressive symptoms → PIU | .43*** | .35** | .29** |
| Impulsivity → PIU | .38*** | .35*** | .36*** |
| Direct effect (c′) | .10 | -.63 | 4.26*** |
| Total effect (c) | -.21* | -2.82*** | 6.17*** |
| Indirect via depressive symptoms | -.17 (-.29,-.06) | -1.15 (-2.05,-.36) | 1.13 (.20,2.29) |
| Indirect via impulsivity | -.08 (-.18,-.01) | -.54 (-1.16,-.10) | .03 (-.77,1.03) |
| Serial indirect effect | -.06 (-.11,-.03) | -.49 (-.92,-.19) | .75 (.29,1.38) |
| Total indirect effect | -.31 (-.47,-.18) | -2.18 (-3.22,-1.29) | 1.91 (.66,3.35) |

*Note. Unstandardized regression coefficients (B) are reported. Values in parentheses represent 95% percentile bootstrap confidence intervals (10,000 resamples). All models controlled for age, gender, and daily personal internet use. The indirect effects represent (1) the pathway through depressive symptoms, (2) the pathway through trait impulsivity, and (3) the serial pathway through depressive symptoms and trait impulsivity. PIU = problematic internet use. *p < .05, **p < .01, ***p < .001.*

**Supplementary Table 5. Direct, Indirect, and Total Associations in the Serial Models With the Socio-Occupational Predictors Mutually Controlled, for the Theory-Specified and the Alternative Order of the Mediators**

| **Effect** | **Model 1 B (95% CI)** | **Model 2 B (95% CI)** | **Model 3 B (95% CI)** |
| --- | --- | --- | --- |
| *Panel A. Theory-specified order: negative affect → impulsivity* | | | |
| X → Negative affect | -.03*** (-.04,-.02) | -.31*** (-.39,-.23) | .35*** (.21,.49) |
| X → Impulsivity | -.13 (-.28,.03) | -.61 (-1.86,.65) | -.53 (-2.56,1.51) |
| Negative affect → Impulsivity | 4.94*** | 4.94*** | 4.94*** |
| Negative affect → PIU | 3.43** | 3.43** | 3.43** |
| Impulsivity → PIU | .33*** | .33*** | .33*** |
| Direct effect (c′) | .18 | -.84 | 4.27*** |
| Total effect (c) | -.00 | -2.63*** | 5.89*** |
| Indirect via negative affect | -.09 (-.18,-.02) | -1.07 (-1.95,-.28) | 1.21 (.29,2.35) |
| Indirect via impulsivity | -.04 (-.12,.02) | -.20 (-.70,.21) | -.18 (-.90,.64) |
| Serial indirect effect | -.04 (-.08,-.02) | -.51 (-.95,-.20) | .58 (.21,1.10) |
| Total indirect effect | -.18 (-.30,-.08) | -1.78 (-2.79,-.90) | 1.62 (.47,2.92) |
| *Panel B. Alternative order: impulsivity → negative affect* | | | |
| X → Impulsivity | -.26** (-.41,-.10) | -2.14*** (-3.32,-.97) | 1.22 (-.83,3.26) |
| X → Negative affect | -.02*** (-.03,-.01) | -.26*** (-.34,-.18) | .32*** (.19,.46) |
| Impulsivity → Negative affect | .02*** | .02*** | .02*** |
| Impulsivity → PIU | .33*** | .33*** | .33*** |
| Negative affect → PIU | 3.43** | 3.43** | 3.43** |
| Direct effect (c′) | .18 | -.84 | 4.27*** |
| Total effect (c) | -.00 | -2.63*** | 5.89*** |
| Indirect via impulsivity | -.09 (-.18,-.02) | -.72 (-1.38,-.23) | .41 (-.29,1.35) |
| Indirect via negative affect | -.07 (-.14,-.02) | -.90 (-1.65,-.23) | 1.11 (.25,2.24) |
| Serial indirect effect | -.02 (-.05,-.003) | -.17 (-.40,-.03) | .10 (-.09,.32) |
| Total indirect effect | -.18 (-.30,-.08) | -1.78 (-2.79,-.90) | 1.62 (.47,2.92) |

*Note. Unstandardized regression coefficients (B) are reported. Values in parentheses represent 95% percentile bootstrap confidence intervals (10,000 resamples). All models controlled for age, gender, daily personal internet use, and the two remaining socio-occupational predictors (Model 1: work engagement and work addiction; Model 2: perceived social support and work addiction; Model 3: perceived social support and work engagement). In Panel A, the indirect effects represent (1) the pathway through negative affect, (2) the pathway through trait impulsivity, and (3) the serial pathway through negative affect and trait impulsivity; in Panel B, (1) the pathway through trait impulsivity, (2) the pathway through negative affect, and (3) the serial pathway through trait impulsivity and negative affect. PIU = problematic internet use. *p < .05, **p < .01, ***p < .001.*
